# Supplementary material for: Are carbon emissions trading and green financial instruments synergistic? -Comprehensive quantitative research based on content analysis
Source: PLoS One. 2024 Mar 7;19(3):e0298601. doi: 10.1371/journal.pone.0298601 (PMC10919601; doi:10.1371/journal.pone.0298601)
Supplement: S1 Data — (ZIP) [file pone.0298601.s001.zip › Data/地级市Z_F工作报告.zip!/╕ú╜¿/╚2├≈╩╨/└┤╫╘╣1⁄2╓┌║┼í╛┬φ┐╦╩2╛▌═°í┐_2017.docx]

各位代表：

　　现在，我代表三明市人民政府向大会作政府工作报告，请予审议，并请市政协各位委员和其他列席人员提出意见。

　　一、过去五年工作回顾

　　刚刚过去的一年，是我市实施“十三五”规划的开局之年。面对复杂的经济形势和繁重的改革发展稳定任务，在省委省政府和市委的坚强领导下，全市各级政府各部门凝心聚力、攻坚克难，深入开展“敢担当、解难题、立新业”和“大干150天，加快推进‘五个一批’项目建设活动”，全力抓好稳增长、调结构、强动力、惠民生、防风险各项工作，经济保持缓中趋稳、稳中向好的发展态势。初步预计，全市生产总值1855亿元，增长7.8%；规模以上工业增加值847.9亿元，增长7.5%；地方一般公共预算收入94.7亿元，增长1.08%；固定资产投资2141亿元，增长12%；外贸出口133亿元，增长13%；实际利用外商直接投资1.71亿美元，增长9.3%；社会消费品零售总额482亿元，增长9.8%；城镇居民人均可支配收入29749元，增长8.6%；农村居民人均可支配收入14023元，增长9.5%；居民消费价格总水平上涨1.1%，实现了“十三五”良好开局，标志着市十二届人大一次会议确定的五年主要目标任务顺利完成。

　　五年来，我们深入学习贯彻习近平总书记系列重要讲话精神和对福建、对三明工作的重要指示，全面贯彻党的十八大和十八届三中、四中、五中、六中全会精神，按照省委省政府和市委决策部署，主动适应经济发展新常态，有效应对经济下行压力，经济保持稳定增长，各项事业全面进步，一些重要领域取得突破：

　　——综合实力明显增强。五年地区生产总值、地方一般公共预算收入年均分别增长9.8%、4.11%，人均生产总值达1.06万美元，固定资产投资累计完成8083亿元，机科院高端机械装备产业园、中节能环保产业园和台明球墨铸管、金明新材料、三农氟化工、月兔空调、翔丰华新能源材料等一批重大产业项目建成投产。

　　——交通条件更加完善。在全省率先实现县县通高速，动车从无到有，三明沙县机场实现通航，三明“陆地港”投入运营，综合交通体系初步形成。

　　——改革创新走在前列。医改、林改、金改等品牌在全国全省打响，国家扶贫改革试验区、全国小微企业创业创新基地城市、三明生态新城国家低碳城等一批国家试点试验任务落地三明。

　　——民生改善成效显著。五年实施为民办实事项目145个，脱贫7.6万人，提前实现“双高普九”和国家义务教育发展基本均衡县创建目标，被评为国家公共文化服务体系示范区、全国创业先进城市，城乡居民人均可支配收入年均分别增长10%、11.3%。

　　——城乡魅力大幅提升。跻身全国文明城市，荣获全国双拥模范城“七连冠”、全国综治“长安杯”，入选全国生态保护与建设示范区、国家森林城市，12个县（市、区）全部列入中央苏区范围，海西三明生态工贸区和生态新城建设取得重要进展。

　　五年来的主要工作是：

　　（一）持续调结构促转型。加快传统产业改造提升，五年实施重点技改项目1658个，累计完成技改投资3245亿元，去年冶金及压延、汽车及机械装备、林产加工、纺织等四大传统主导产业产值突破1900亿元。着力培育壮大战略性新兴产业，坚定不移推进资源往产业走，稀土、氟化工、石墨（烯）、生物医药等新兴产业发展势头良好，高技术产业增加值年均增长18.4%，三明高新技术产业开发区升格为国家级高新区。加快发展现代农业，实施千亿现代农业产业跨越发展行动计划，大力发展高优粮食、绿色林业、精致园艺、生态养殖和现代烟草等5大产业，现代特色农业产值突破1000亿元，三明现代农业科技示范园被列入国家农业科技园区。大力发展现代服务业，电子商务、现代物流、旅游、金融、健康养老等产业加快发展，新增3个创建国家全域旅游示范区、2个国家地质公园、1个国家级自然保护区，实现省级以上电子商务进农村示范县全覆盖，新引进5家区外银行，服务业增加值年均增长8.8%。强化创新驱动发展，新建机科院海西分院等6家“国字号”研发中心，新设院士专家工作站18家。三次产业比重由16.1∶50.4∶33.5调整为15.3∶49.7∶35。

　　（二）持续建城乡美环境。加快推进中心城市建设，科学编制三明市城市总体规划，205国道市区过境线、三明南站、万达广场、碧桂园等项目顺利实施，快速通道一期和市区“六馆一场一宫”、城市绿道一二期、第二供水工程建成投入使用，市区建成区面积从27.85平方公里拓展到37.45平方公里；启动三明生态新城建设，三明陆地港、电商园、职教园、湿地公园等一批项目建成，有效拓展了市区发展空间。大力发展县域经济，永安、沙县入选全省县域经济实力“十强”县，大田、建宁入围全省县域经济发展“十佳”县，5个省级扶贫开发工作重点县发展步伐加快。稳步推进新型城镇化建设，永安、大田分别列入国家、省级新型城镇化试点，尤溪洋中镇被确定为省级“小城市”培育试点，明溪药谷小镇、永安石墨小镇入选全省首批特色小镇，一批美丽乡村成为旅游热点。积极推进生态文明建设，五年完成造林绿化面积196万亩、综合治理水土流失283万亩，县级以上饮用水源水质达标率100%，辖区三条主要水系省控断面水质合格率100%，市区空气质量达到国家二级标准；三明成为省级生态市，6个县成为国家生态县，87%的乡镇成为国家级生态乡镇，泰宁入选首批国家主体功能区建设试点并纳入国家重点生态功能区。全市城镇化率从51.1%提高到56.3%。

　　（三）持续抓改革增活力。深化医改，实行“三医联动”和“四级联推”医药卫生体制改革，中央深改组第21次会议专门听取三明医改情况汇报，全国卫生与健康大会指定典型发言，“三医联动”“两票制”等改革做法在全国推广。推进林改，在全国率先推行林权按揭贷款、林业互联网金融等产品，全市累计发放林权抵押贷款102亿元。探索金融改革，沙县农村金融改革和园区企业资产按揭贷款经验做法在全省推广，新增3家企业在境内外上市、6家企业在“新三板”挂牌，发行公司债、企业债总额超过60亿元。深化商事制度改革，推行“多证合一”。稳妥推进农村改革，探索形成土地入股、土地托管等多形式土地流转模式，沙县建成全省首个县级农村产权交易中心。健全生态保护机制，建立“河长制”，排污权交易、企业环境信用评价、环保责任保险三项改革得到中央深改办肯定，大田县生态综合执法项目获第四届中国法治政府奖提名奖。深化财政体制改革，推进预算管理制度和国库集中支付改革，全面推开营改增试点。进一步扩大开放，外贸进出口额累计104亿美元，实际利用外资累计到资6.9亿美元；积极承接自贸区外溢效应，推进电子口岸建设，进口商品直销中心、“陆地港”保税仓库等项目建成运营；深化明港澳台侨合作，举办世界客属第25届恳亲大会和第七届海峡两岸客家高峰论坛，把“11·6”林博会办成“天天林博会”。

　　（四）持续惠民生保和谐。五年累计财政民生支出807.7亿元，占财政总支出78.2%。五年累计实现新增城镇就业13.2万人、农村劳动力转移就业22.25万人。五年建设保障性住房3.42万套、竣工2.2万套，完成造福工程搬迁和危房改造3.9万户、16.5万人。稳步扩大社会保险覆盖面，城乡社会救助和应急灾害救助体系不断完善，困难群众基本生活得到保障。优先发展教育事业，义务教育标准化学校建设完成率100%，中小学生“幸福成长工程”经验做法在全省推广。加强卫生和人口计生工作，医疗卫生资源总量快速增长，“诚信计生·幸福家庭”创建全面推进。大力发展文化事业和文化产业，4个县被评为全国文化先进县，文化产业增加值增幅保持全省前列。广泛开展全民健身活动，成功承办全国首届青年运动会乒乓球赛事，三明籍运动员邓薇、柯丽婷分别勇夺我市首枚奥运会、残奥会金牌。深入推进群众性精神文明建设，全国“五讲四美三热爱”30周年研讨会在我市召开。有效应对泰宁开善泥石流等严重自然灾害，进一步建立健全科学高效的防灾减灾机制。创新社会综合治理，强化信访工作，抓好安全生产，有效保障食品药品安全，社会保持安定稳定。老龄、工会、青少年、妇女儿童、残疾人工作扎实开展，司法行政、统计、科普、民族宗教、档案、地方志、水文、气象、防震、库区移民等工作取得新进展。人民防空、民兵预备役、国防后备力量建设得到加强。

　　（五）持续优服务转作风。深入开展党的群众路线教育实践活动、“三严三实”专题教育和“两学一做”学习教育，严格落实中央八项规定精神，驰而不息反对“四风”。全面推进依法行政，法治政府建设取得新成效。认真执行人大及其常委会决议决定，依法接受法律监督和工作监督，认真办理人大代表意见建议。自觉接受人民政协民主监督，开展多形式协商，认真办理政协提案。推进简政放权，市级行政审批事项从543项精简到214项，推行“六张清单”制度，在全省率先设立市级中介服务中心。强化政府绩效管理，机关效能建设不断加强。开展“千名干部服务千家企业”活动，推动干部到一线服务项目、服务企业。健全完善政务公开，加强行政监察和审计监督，党风廉政建设和反腐倡廉工作深入推进。

　　各位代表，过去五年成绩来之不易，这是省委省政府和市委正确领导的结果，是全市人民团结奋斗的结果，是社会各界大力支持的结果。在此，我代表市人民政府，向为三明建设发展付出辛勤劳动的全市人民，向给予政府工作有力支持与有效监督的人大代表和政协委员、各民主党派、工商联、各人民团体、无党派人士、离退休老同志及社会各界人士，向积极发挥作用的中央和省属驻明单位、驻明部队、武警官兵、政法干警，向关心支持三明发展的港澳台同胞、海外侨胞、国际友人，致以衷心的感谢和崇高的敬意！

　　在看到成绩的同时，我们也清醒地认识到，三明经济社会发展仍面临不少困难和问题，主要是：经济下行压力较大，部分产业和企业发展仍较困难，财政增收比较乏力，信贷风险防控难度加大；高新技术产业比重偏低，企业创新能力不强，新旧动能接续转换仍需时日；投资增长后劲不足，支撑经济转型发展的大项目好项目不够多，民间投资和制造业投资增速下降；民生社会事业领域还存在短板，与群众期盼还有差距；有的干部办事效率、服务水平有待提高，不作为、慢作为等现象在部分单位和领域仍有存在，等等。对于这些问题，我们一定认真对待，创新举措，切实加以解决。

　　二、新一届政府总体要求、主要目标和重点任务

　　今后五年，是全面落实“十三五”规划、全面建成小康社会的关键时期，任务繁重、使命光荣。政府工作的总体要求是：高举中国特色社会主义伟大旗帜，以邓小平理论、“三个代表”重要思想、科学发展观为指导，深入贯彻以习近平同志为核心的党中央治国理政新理念新思想新战略，紧紧围绕“五位一体”总体布局和“四个全面”战略布局，牢固树立和落实五大发展理念，主动融入全省“再上新台阶、建设新福建”发展大局，按照市第九次党代会部署，突出“念好发展经、画好山水画”工作主题，坚持稳中求进总基调，着力提高发展质量和效益，着力创新驱动和供给侧结构性改革，着力改善民生和创新社会治理，着力加强生态文明建设和环境保护，推动各项事业发展再上新台阶，奋力建设更富活力更具实力更有竞争力的新三明。

　　主要目标是：在提前实现地区生产总值和城乡居民收入比2011年翻一番的基础上，力争到2021年地区生产总值、一般公共预算总收入分别突破2800亿元和200亿元。

　　重点任务是：

　　——推动创新发展再上新台阶。把创新作为引领发展的第一动力，实施创新驱动战略，强化产业创新，深入推进供给侧结构性改革，力争全市农林牧渔业总产值超520亿元、规模以上工业增加值超1500亿元、服务业增加值超1100亿元；强化企业创新主体地位，加快建设一批科研创新平台，深入实施“海纳百川”高端人才聚集计划，高新技术产业增加值占地区生产总值的比重达13%。强化改革创新，“三医联动”改革、集体林权制度改革继续保持全国领先地位，农村产权制度改革、投融资体制改革稳步推进，不断增强发展动力和活力。

　　——推动协调发展再上新台阶。围绕构建以三明中心城市为核心、县域城市为骨干、特色小镇为支撑、美丽乡村为基础的城镇化新格局，推进市区与沙县、永安同城化进程，全力打造区域性中心城市；提升县域经济特色，按照“一县一园区”方向，引导各地融入全市发展大局和产业布局，走差异化发展的路子；推进小城镇综合改革建设，打造一批“产、城、人、文”四位一体有机结合的特色小镇；推进美丽乡村建设，落实“六好六美”标准，保护历史文化名镇、名村和传统村落，培育一批美丽乡村。力争到2021年常住人口城镇化率达65%。

　　——推动绿色发展再上新台阶。坚持“绿水青山就是金山银山”理念，推进生态文明制度建设，落实国家生态文明试验区福建方案涉及三明的改革试验任务，努力推出更多可复制、可推广的工作机制。推进绿色低碳循环发展，落实产业准入负面清单，突出发展节能环保的战略性新兴产业，积极谋划核电、抽水蓄能、风电等绿色能源。落实主体功能区规划布局，实行能源和水资源消耗、建设用地等总量和强度双控，抓好三明生态新城国家低碳城试点建设。推进生态环境保护，落实大气、水、土壤污染防治行动计划，加强生态系统修复保护，倡导绿色发展方式和生活方式，建设天更蓝、地更绿、水更净、环境更友好的美丽家园。

　　——推动开放发展再上新台阶。努力构建立足两岸、服务全国、面向世界的开放格局，深入对接福建自贸试验区建设，加强与港澳台地区交流。努力构建充满活力、富有效率的开放环境，加快推进“两纵两横”铁路网、“一纵三横四联”高速公路网、“四纵三横三联”国省干线骨架网建设，实现县县通铁路；拓展三明“陆地港”功能，推行外商投资负面清单管理模式。努力构建“双向”投资、合作共赢的开放路径，积极融入“一带一路”，支持行业优势企业加快“走出去”，把更多有实力的企业“引进来”。

　　——推动共享发展再上新台阶。打好精准扶贫攻坚战，全面推进国家扶贫改革试验区建设，支持5个省级扶贫开发工作重点县加快发展，力争在20l7年完成现行国定扶贫标准贫困人口脱贫任务，巩固一年后确保实现全部脱贫；在2019年完成省定扶贫标准贫困人口脱贫任务，巩固一年后确保建档立卡贫困村、贫困空壳村、市级扶贫开发重点乡镇、省级扶贫开发工作重点县实现全部脱帽。推进基本公共服务均等化，坚持量力而行、尽力而为，着力解决就业、教育、社会保障、文化体育、医疗卫生、健康养老等人民群众关心关注的问题。广泛开展群众性精神文明创建活动，持续推进“平安三明”建设，加强和创新社会治理，努力建设法治三明，始终保持社会和谐稳定。

　　三、2017年主要工作

　　2017年是实施“十三五”规划的重要一年，是供给侧结构性改革的深化之年。我们将坚持稳中求进总基调，适应把握引领经济发展新常态，以提高发展质量和效益为中心，以供给侧结构性改革为主线，以振兴实体经济为重点，突出产业升级，突出项目带动，突出改革攻坚，突出民生保障，突出风险防范，促进经济平稳健康发展和社会和谐稳定。今年我市经济社会发展主要预期目标是：地区生产总值增长8.5%；地方一般公共预算收入增长5%；固定资产投资增长15%；外贸出口增长3%；实际利用外商直接投资增长6%；城镇登记失业率控制在3.8%以内；社会消费品零售总额增长11%，居民消费价格涨幅3%左右；城镇居民人均可支配收入增长8.5%，农村居民人均可支配收入增长9%；完成单位生产总值能耗降低和主要污染物减排年度任务。围绕上述目标，重点做好以下六个方面工作：

　　（一）推进供给侧结构性改革，加快产业转型升级

　　深化“三去一降一补”。落实省上下达的去产能任务，坚决淘汰一批落后产能，坚决防止已关闭企业“死灰复燃”。落实好房地产各项政策措施，指导各地因城因地发展房地产，促进房地产市场平稳健康发展。加大企业帮扶力度，扩大股权融资规模，强化企业债券杠杆约束，降低企业杠杆率；落实企业减费措施，进一步降低用能、物流等要素成本和制度性交易成本；引导企业用好用足惠企政策，扶持做大现有龙头企业，帮助协调解决各类发展要素问题。坚持既补硬短板也补软短板、既补发展短板也补制度短板，重点围绕基础设施、产业发展、社会事业和民生保障等领域短板，继续策划实施一批投资工程包项目。

　　推动制造业中高端发展。突出发展战略性新兴产业，培育壮大氟化工、石墨（烯）、稀土、生物医药等新兴产业，加快推进海斯福高端含氟材料、锂离子正极材料二期、石墨负极材料、汇天药业扩建等重点项目，力争实现产值620亿元以上。改造提升冶金及压延、汽车及机械装备、林产加工、纺织等4个传统产业，支持龙头企业增资扩股、兼并重组，力争实现产值2000亿元以上。实施新一轮技术改造专项行动计划，完成技术改造投资800亿元以上，抓好310项重点技改项目建设，统筹推进“两化”融合、智能制造。整合提升15个重点工业园区，加快石墨（烯）产业园、氟化工专业产业园、信息经济产业园、生物医药产业园等特色专业园区建设，积极开展园区“二次招商”，完成固定资产投资220亿元以上，规模以上工业总产值突破1400亿元。

　　促进现代服务业全面发展。加快发展商贸物流、休闲旅游、健康养老三大主导产业，推进全国物流标准化试点城市和三明陆地港冷链物流、海鑫钢材物流配送等项目建设，力争新增冷链库容2万吨、限上商贸企业30家以上；推进泰宁、尤溪、建宁创建国家全域旅游示范区，大力发展乡村民宿、汽车露营地等旅游新业态；推进医养结合试点城市建设，打造一批健康养老基地。积极培育电子商务、创意设计、文化体育三大先导产业，推进10个省级以上电子商务进农村示范县、56个文化产业重点项目建设，加快竞赛表演、健身休闲等体育服务业发展，文化产业增加值达55亿元。提升发展金融、服务外包、科技服务三大配套产业，拓展服务外包行业领域，推广园区企业资产按揭贷款、小微企业助保贷等业务，力争年内新增贷款130亿元。

　　加快发展特色现代农业。深入推进农业供给侧结构性改革，把增加绿色优质农产品供给放在突出位置。持续壮大高优粮食、绿色林业、精致园艺、生态养殖、现代烟草5大产业20条产业链，扶持壮大龙头企业，强化品牌创建和经营，新增“三品一标”农产品20个以上。加强现代农业“一区三园”建设，大力推广农业“五新”，加快“中国稻种基地”建设，新增设施农业面积2000亩以上。加强粮食产能区和高产示范片建设，确保粮食播种面积稳定在320万亩、总产量稳定在110万吨以上。大力培育家庭农（林）场、专业大户、农民合作社等新型经营主体，因地制宜发展农业观光、农家乐等休闲农业。积极发展“互联网+农业”，鼓励发展定制农业、众筹农业和农产品线上销售，不断衍生农业新业态，推进一二三产融合发展。

　　（二）实施创新驱动战略，推进创新型城市建设

　　加大产业创新力度。加强产业研发平台建设，在汽车及零部件、铸锻工艺及设备、机械装备等领域提升一批工程（技术）研究中心，在高端纺织、氟化工、石墨新材料和生物医药等产业再建一批产业研发平台。强化企业自主创新，力争新增省级以上重点（工程）实验室、工程技术研究中心、企业技术中心5个以上，新增院士工作站10家以上，新增国家高新技术企业15家以上。加快实施科技小巨人领军企业行动计划，精准帮扶种子期、初创期、成长期的企业发展，新增科技小巨人领军企业20家以上。

　　深化大众创业万众创新。加快全国小微企业创业创新基地城市建设，培育“一县一特色”双创示范基地，探索形成更多可复制的示范经验，确保通过国家考核验收。注重“双创”平台建设，加快打造一批低成本、开放式的众创空间、星创天地，提高入驻企业创业创新成功率；支持社会力量创办中介服务机构，培育一批区域性中小企业服务中心、创新驿站等服务机构。健全创新创业服务和政策支持体系，鼓励科技人员、大学生、返乡农民工等重点人群创新创业，新增小微企业4200家以上。

　　完善创新体制机制。建立财政性科技投入稳定增长机制，落实好高新技术企业所得税减免、研发费用加计扣除等税收优惠政策。积极引进风险投资公司，鼓励开展专利权质押贷款、科技保险等业务，促进科技与金融协同创新。落实企业研发、购买和使用首台（套）产品奖励政策，提高科技成果完成人和作出重要贡献人员的奖励比例。深入实施“海纳百川”高端人才聚集计划，建立“星期天工程师”“假日教授专家”等柔性引才机制，重视发挥本地人才作用。加大知识产权保护和利用力度，注重科普工作实效，营造鼓励创新的良好氛围。

　　（三）强化项目带动，积极扩大有效投资

　　加快重点项目建设。全力抓好1005个、总投资6403亿元的“五个一批”项目。着力抓好464个列入省行动计划重大项目，全年完成投资450亿元以上。突出重大基础设施项目建设，全面推进浦梅铁路建宁至冠豸山段、兴泉铁路、莆炎高速永泰梧桐至尤溪中仙段建设，确保年内南龙铁路、厦沙高速和三明西互通建成通车，莆炎高速尤溪至建宁段全线开工建设，续建和新开工国省干线197公里；开工建设生态工贸区安全生态水系示范区项目，加快14个中型水库及烟基水源工程建设，推进闽江上游重点流域防洪工程；新建、改造城市道路和地下管网500公里以上，全市新增城市公共停车位1500个，推进海绵城市建设；抓好海西天然气管网三明段一期、泰宁池潭电厂扩建等项目建设，推进农村电网改造升级工程。突出重大产业项目建设，实施200个以上总投资超5000万元工业重点项目，着力推进三钢生产线升级改造及物联云商、青山纸业超声波制浆、建新轮胎二期、住宅产业化等项目开工建设。大力挖掘投资潜力，加快策划和实施一批国家专项建设基金项目。

　　健全项目推进机制。开展“五比五晒”项目竞赛活动，落实“五个一批”项目工作机制，加强项目策划储备和动态管理，实现项目持续滚动发展。完善重点项目“一月一协调、一季一督查、一季一通报”和重大项目挂牌服务机制，强化各类要素保障，增强项目建设实效。继续实施一批经营性项目，发挥国有投资支撑引领作用。落实鼓励民营投资政策，促进更多社会资本投向基础设施、公共服务领域。

　　强化招商引资工作。拓展深化与机科院、中节能的合作，持续加强与中化集团、中核集团、中纺院、国机集团等央企对接，力争取得新突破。推进与省属企业、厦门企业对接合作，力争年内新对接签约项目投资额230亿元以上。深化民企对接，实施“明商回归”工程，确保对接民企合同项目投资额300亿元以上。紧盯世界500强和台湾百大企业、港澳侨大型企业，用好“9·8”投洽会、“11·6”林博会等平台，力争新批办外资项目增长12%。

　　（四）着力壮大区域经济，促进城乡协调发展

　　做优做强市区经济。全面落实《市区经济重点项目建设行动计划》，健全八个重点项目工作组推进机制，着力实施207个市区经济重点项目，增强市区的凝聚力、向心力和带动力。推动市区产业提升，统筹推进9个特色产业园建设，抓好72个工业重点项目，力争年内市区工业增加值占全市比重提升1～2个百分点；推进徐碧商务区、海鑫市场群、三明郊野国家地质公园等现代服务业项目建设，提升梅列中心商贸区、三元产业配套商贸区发展水平；加快发展都市现代农业，着力打造“一园二带三基地”。推动市区城建提效，全面推进中心城市路网建设，205国道市区过境线全线建成通车，尽快形成市区公路外环，促进市区与各开发区互联互通；有序推进北部新城、南部新城等重点片区开发，开展市区生态修复、城市修补，抓好18项重点城建项目。推动市区社会事业提质，教育方面重点推进优质教育扩容工程，支持普通高中优质发展，开展与厦门名牌学校交流合作；医疗卫生方面重点推进市属医疗机构与省内外高水平医院合作办医，加快市第一医院外科病房综合楼、市妇幼保健院等项目建设；文化体育方面重点提升一批街道社区综合文化服务中心，积极组织品牌赛事，提高市区集聚人口能力。

　　壮大县域特色经济。支持县域发展特色产业，鼓励各地依托特色资源，走差异化发展路子；支持永安、沙县发挥引领带动作用，持续争创全省县域经济实力“十强”县；支持尤溪、大田、将乐壮大经济总量、提升发展质量，争创全省县域经济发展“十佳”县；支持明溪、清流、宁化、建宁、泰宁加快脱贫步伐，补齐发展短板，增强自我发展能力。深入实施“大城关”战略，抓好县城规划和建设，有序推进新区开发和旧城改造，推动县城向区域中小城市提升。深化全市国家中小城市综合改革试点和尤溪洋中镇省级“小城市”培育试点，推进永安国家级、大田省级新型城镇化试点建设，全面实施居住证制度，加快基本公共服务向常住人口覆盖，引导人口向城市和建制镇集聚。

　　优化美化城乡环境。贯彻实施《三明市城市市容和环境卫生管理条例》，深化全国文明城市建设，推广“街长制”“微城管”，完善公交网络系统、建设公共自行车系统，开展交通拥堵综合整治，加强社区建设与管理，提升城市精细化管理水平。深化城乡宜居环境建设行动，整治提升城镇“三边三节点”，实施农村人居环境治理工程，推进美丽乡村建设。深化水流域环境综合治理，强化畜禽养殖面源污染整治，加强饮用水水源保护；推进大气污染防治，突出重点企业治理工程建设，深化扬尘面源治理；加强土壤环境保护与治理，实施山水林田湖生态保护和修复工程，完成造林绿化和森林经营222万亩，综合治理水土流失20万亩。

　　加快生态新城建设。积极推进三明生态工贸区建设，继续以“十同”为重点，加快市区与沙县、永安同城化步伐。大力推进生态新城“一带六区”建设，抓好国家级现代农业科技示范园、国家级高新技术产业园区、国家低碳城试点建设。全面推进生态新城核心区建设，做好总体城市设计工作，重点实施湿地公园三期、商业文化中心、水上乐园、商业综合体等项目，加快医养特色小镇、三甲医院规划建设，构建宜居宜业的中央商务区；抓紧陆地港专业市场、电商产业园三期等项目建设，大力发展保税延展、保税展示交易和跨境电商业务，促进商贸金融产业发展；加快职教园二期、北大附属实验学校、新城小学建设，打造优质教育体系。

　　（五）深入推进改革开放，增创体制机制优势

　　深化重点领域改革。持续深化“三医联动”改革，探索全民健康四级共保新机制，健全城乡居民健康档案，努力为群众提供更高水平的健康服务和保障。持续深化林改，创新普惠制林业金融，力争全市新型林业经营主体覆盖面达55%，新增林权抵押贷款10亿元，林权交易面积达20万亩。持续深化金融改革，引导银行机构加大对实体经济支持力度，加强信贷风险防范和处置工作，探索发行绿色债券，鼓励和支持企业上市，做大直接融资规模。推进农村承包土地经营权抵押贷款试点，探索农村林、地、房“三权”抵押贷款业务，激活农村产权资源。开展国资国企改革，推进国有资产重组和国有企业分类改革，做强市属国有企业。深入推进城市执法体制改革，改进城市管理工作。全面落实国家生态文明试验区18项年度改革任务，巩固提升“河长制”，建立生态保护执法、司法双保险机制，持续抓好全国生态保护与建设示范区工作。

　　积极扩大对外开放。持续承接自贸区溢出效应，复制推广自贸区试验创新成果，拓展陆地港口岸和保税仓储功能，提升对外开放和口岸通关便利化水平。稳定外贸增长，力争培育年出口总额超千万美元企业75家以上；主动融入“海丝”核心区建设，推动我市钢铁、机械装备、汽车、水泥等优势产业产品“走出去”，力争对“一带一路”沿线区域出口增长10%以上。扩大对外交流交往，深化明台交流合作，密切与港澳地区、国外友城的沟通联系，加强与海外华侨华人及其社团的友好往来。

　　（六）注重保障改善民生，扩大基本公共服务

　　打好精准扶贫攻坚战。深入推进国家扶贫改革试验区建设，统筹用好老区苏区政策和扶贫开发专项资金，构建专项扶贫、行业扶贫、社会扶贫互为补充的大扶贫格局。拓展提升“348”精准扶贫工作机制，实施精准扶贫“九个一批”和“五项工程、三大行动”，大力开展产业扶贫，因地制宜开展乡村旅游扶贫、光伏扶贫、电商扶贫等，扩大扶贫小额贷款覆盖面，深化扶贫资金量化折股、资源资产入股分红、保险扶贫等改革试点，确保全年1.7万人脱贫。推进造福工程易地搬迁，全年实施造福工程1.03万人。支持少数民族乡村加快发展，继续加强库区移民安置和后期扶持工作。

　　全面加强社会保障。落实就业创业各项扶持政策，统筹做好农民工、高校毕业生、就业困难人员等重点群体就业工作，新增城镇就业1.9万人以上。加大社会保险扩面力度，推进基本养老保险法定人员全覆盖。稳步提高城镇企业退休职工养老保险待遇和城乡居民基础养老金，进一步提高优抚对象抚恤和生活补助、城乡低保补助水平。加大棚户区改造力度，实施棚户区改造4660套，基本建成保障性安居工程住房2200套。加快完善养老服务体系，推进医养融合发展，打造“15分钟社区居家养老服务圈”。健全未成年人、农村留守儿童、妇女、残疾人关爱服务体系，大力发展社会福利、社会救助、红十字会、慈善、老龄事业，支持关心下一代工作。

　　加快发展社会事业。推进学前教育三年行动计划，普惠性学前教育覆盖面扩大到75%；全面改善义务教育薄弱学校基本办学条件，全面推广中小学生“幸福成长工程”经验，全面推进“教育强县”创建工作，促进义务教育均衡发展；深化普通高中质量提升工程，力争再创高考佳绩；大力促进职业教育发展，推进“二元制”和现代学徒制人才培养模式改革试点；支持三明学院争创国家示范性应用型本科高校，推动三明医学科技职业学院、福建水利电力职业技术学院争创省示范性现代职业院校，促进校市融合发展；支持特殊教育、民族教育、终身教育、民办教育发展。巩固提升国家公共文化服务体系示范区创建成果，完善公共文化服务“六联六创”机制，推进基层综合性文化服务中心建设；深入挖掘客家文化、朱子文化、闽学文化等特色文化内涵，推动红色文化遗址保护立法，抓好万寿岩国家考古遗址公园建设。加强计划生育工作，完善医疗卫生服务体系，加强产科、儿科、精神科等薄弱专科建设，强化疾病预防控制，提升突发公共卫生事件应急处置能力，持续开展爱国卫生运动。落实全民健身实施计划，积极发展老年体育，培养竞技体育后备人才，做好参加第十三届全运会服务保障工作。

　　加强和创新社会治理。加快推进法治三明建设，深入开展“七五”普法。全面加强社会信用体系建设，强化守信激励和失信惩戒。完善群众性精神文明建设长效机制，大力弘扬三明精神，打响“为文明点赞，为三明点赞”品牌。深化“平安三明”建设，建立立体化治安防控体系，扎实做好社会稳定工作。全面落实信访工作责任制，持续推进阳光信访、责任信访、法治信访建设，依法公开处理信访事项。强化食品药品监管，健全食品药品追溯、监测体系。完善和落实安全生产责任制，深化重点行业领域专项整治，坚决遏制重特大安全生产事故发生。加强应急处置能力建设，提高防灾减灾救灾水平。密切与各民主党派、工商联、无党派代表人士的联系与协商，支持工会、共青团、妇联等群团组织发挥更大作用，做好外事侨务、民族宗教工作。深化双拥共建，加强国防动员、国防后备力量和人民防空建设，推进军民融合发展。

　　四、加强政府自身建设

　　我们要牢固树立政治意识、大局意识、核心意识和看齐意识，坚决维护以习近平同志为核心的党中央权威，更加扎实地把党中央、国务院和省委、省政府及市委的各项决策部署落到实处。

　　（一）注重务实。坚持深入调查，摸清实际情况，认真贯彻落实上级部署，针对性研究出台政策措施，增强主动性、减少盲目性、克服片面性。坚持问题导向，多到贫困偏远的农村去，多到矛盾集中的地方去，多到项目建设的现场去，及时协调解决困难和问题，努力为基层和企业排忧解难。坚持一抓到底，弘扬“滴水穿石”“钉钉子”精神，撸起袖子加油干，对各项工作建立明细账，画好施工图，倒排时间表，立下军令状，确保干一件成一件。坚持循序渐进，树立“功成不必在我”发展观，坚决克服急功近利、短期行为，多做打基础、利长远、惠民生的事，扎扎实实把一张蓝图画到底。

　　（二）注重高效。落实“马上就办”，对作出的部署、决定的事项、安排的项目，做到雷厉风行抓执行，立说立行抓落实，力争最短时间见成效。提高服务效率，深化“放管服”改革，探索运用“互联网+政务”模式，进一步完善网上办事大厅，推进行政服务规范化、标准化建设，实施行政审批“三集中”，让群众少跑腿、项目早落地。强化效能督查，健全常态化督查问责机制，着力整治懒政怠政为官不为，对效率低、服务差、企业和群众意见大的，该问责的严肃问责，坚决消除“中梗阻”，打通工作落实“最后一公里”。

　　（三）注重创新。践行新理念，把五大发展理念始终贯穿落实到政府工作全过程、各环节。激发新活力，进一步解放思想，更新观念，坚决冲破一切妨碍发展的思想观念，坚决改变一切束缚发展的做法规定，坚决革除一切影响发展的体制弊端。探索新办法，深化“敢担当、解难题、立新业”和“千名干部服务千家企业”活动，建立健全正向激励机制和容错纠错机制，鼓励干部敢于负责、勇于创新、善于攻坚，在破解难题中推进发展。追求新标准，把工作放到更高位置来谋划，对标先进地区，借鉴先进经验，强化“比学赶超”，力求各项工作有特色、创品牌、争一流。

　　（四）注重廉洁。坚决推进全面从严治党，严格执行党内政治生活若干准则、廉洁自律准则和纪律处分条例、问责条例、党内监督条例，持之以恒落实中央八项规定精神。坚持依法行政，严格依照法定权限和程序行使权力、履行职责，自觉接受人大及其常委会的法律监督、工作监督和政协的民主监督，主动接受社会监督和舆论监督。深入推进政务公开，实现决策、执行、结果公开透明。加强行政监察，发挥审计监督作用，对重要领域、关键岗位严加监管。健全完善惩治和预防腐败体系，严肃查处违纪违法案件，努力打造风清气正的廉洁政府。

　　各位代表，蓝图已绘就，奋进正当时。让我们更加紧密团结在以习近平同志为核心的党中央周围，在省委省政府和市委的领导下，不忘初心、继续前进，艰苦奋斗、真抓实干，奋力建设更富活力更具实力更有竞争力的新三明，以优异成绩迎接党的十九大胜利召开！
